# Ancestry-Associated Performance Variability of Open-Source AI Models for *EGFR* Prediction in Lung Cancer

**DOI:** 10.1001/jamaoncol.2025.6430

**Published:** 2026-02-12

**Authors:** Mehrdad Rakaee, Amin H. Nassar, Masoud Tafavvoghi, Falah Jabar, Elias Bou Farhat, Elio Adib, Sigve Andersen, Lill-Tove Rasmussen Busund, Mette Pøhl, Åslaug Helland, Alexander Gusev, Biagio Ricciuti, Lynette M. Sholl, Tom Donnem, David J. Kwiatkowski

**Affiliations:** 1 Department of Medicine, Brigham and Women’s Hospital, Harvard Medical School, Boston, Massachusetts; 2 Department of Cancer Genetics, Oslo University Hospital, Oslo, Norway; 3 Department of Medical Oncology, Yale Cancer Center, Yale New Haven Hospital, New Haven, Connecticut; 4 Department of Surgery and Cancer, Imperial College London, London, United Kingdom; 5 Department of Clinical Pathology, University Hospital of North Norway, Tromso, Norway; 6 Department of Clinical Medicine, UiT The Arctic University of Norway, Tromso, Norway; 7 Department of Oncology, Copenhagen University Hospital, Rigshospitalet, Copenhagen, Denmark; 8 Department of Data Science, Dana-Farber Cancer Institute, Boston, Massachusetts; 9 Department of Medical Oncology, Dana-Farber Cancer Institute, Boston, Massachusetts; 10 Department of Pathology, Brigham and Women’s Hospital, Harvard Medical School, Boston, Massachusetts

## Abstract

**Importance:**

Artificial intelligence (AI) models are emerging as rapid, low-cost tools for predicting targetable genomic alterations directly from routine pathology slides. Although these approaches could accelerate treatment decisions in lung cancer, little is known about whether their performance is consistent across diverse patient populations and tissue contexts.

**Objective:**

To evaluate the performance and generalizability of 2 open-source AI pathology models for predicting *EGFR* mutation status in lung adenocarcinoma (LUAD) across independent cohorts and ancestral subgroups.

**Design, Setting, and Participants:**

This cohort study included patients with LUAD from 2 cohorts: Dana-Farber Cancer Institute (DFCI) from June 2013 to November 2023, and a European-based trial (TNM-I) from August 2016 to February 2022. All patients had paired next-generation sequencing data and hematoxylin-eosin–stained whole-slide images. In the DFCI cohort, genetic ancestry was inferred using germline genotype data. Data analyses were performed from July 2025 to September 2025.

**Main Outcomes:**

The primary outcome was model performance for predicting *EGFR* mutation status, measured as the area under the receiver operating characteristic curve (AUC), evaluated overall and across ancestry subgroups and sample types.

**Results:**

Overall, 2098 patients with LUAD were included (mean [SD] age, 66.6 [10.3] years; 1315 female individuals [63%] and 783 male individuals [37%]). In the DFCI cohort (n = 1759; 54 African, 101 American, 95 Asian, 1465 European), *EGFR* mutations were detected in 432 patients (25%). One AI-pathology model achieved an AUC of 0.83 (95% CI, 0.81-0.85) compared with 0.68 (95% CI, 0.65-0.70) for the other model. In the TNM-I cohort (n = 339), *EGFR* mutations were detected in 50 patients (15%), with AUCs of 0.81 (95% CI, 0.74-0.88) and 0.75 (95% CI, 0.68-0.83), respectively. In ancestry-stratified analyses of the DFCI cohort, AUCs for the higher-performing model were 0.84 (95% CI, 0.81-0.86) in patients of European ancestry, 0.85 (95% CI, 0.72-0.94) in African ancestry, and 0.68 (95% CI, 0.55-0.78) in Asian ancestry. In sample type analyses, performance declined in pleural (AUC, 0.66; 95% CI, 0.56-0.76) compared with lung specimens (AUC, 0.86; 95% CI, 0.83-0.88). AI-guided triage analyses showed a potential 57% reduction in rapid *EGFR* testing, while maintaining sensitivity of 0.84 and specificity of 0.99.

**Conclusions:**

This cohort study found that AI-based pathology tools may serve as preliminary adjuncts for *EGFR* prediction in lung cancer, though performance differences by ancestry warrant careful interpretation.

## Key Points

**Question:**

Do open-source artificial intelligence (AI) models for predicting *EGFR* mutations from pathology slides perform consistently across patient populations and clinical settings?

**Findings:**

In this multicohort study of 2098 patients with lung adenocarcinoma from the US and Europe, open-source AI approaches achieved high accuracy for *EGFR* prediction and demonstrated overall robust performance. Subgroup analyses revealed lower accuracy in Asian patients and pleural tissue samples.

**Meaning:**

AI-based histology tools show strong potential as rapid, low-cost adjuncts for identifying *EGFR* mutations; broader validation and recalibration across diverse populations and tissue types will help ensure equitable clinical adoption and maximize their impact in cancer care.

## Teaser

This cohort study evaluated the performance and generalizability of 2 open-source artifical intelligence models in predicting mutations in *EGFR* genes in lung adenocarcinomas using data from 1 US and 1 European cohort.

## Introduction

Deep learning methods have transformed digital pathology by enabling the prediction of molecular biomarkers and clinical outcomes directly from hematoxylin-eosin (H&E) whole-slide images (WSIs).^1,2^ For molecular prediction tasks, such approaches could provide a rapid and inexpensive complement to genetic testing, but their ability to generalize across institutions and patient populations is uncertain. Variability in staining protocols, scanning platforms, and patient demographics including ancestry may contribute to inconsistent performance.^3,4^

*EGFR* mutations are present in approximately 15% to 25% of lung adenocarcinomas (LUAD),^5^ though the frequency varies by geographic region. Accurate determination of *EGFR* mutation status is critical for optimal choice of therapy in both early and advanced stages. However, routine genetic tests (such as polymerase chain reaction– and next-generation sequencing–based) can be limited.^6^ For example, in a recent global survey across 90 countries, more than 40% of clinicians reported treating patients before biomarker results were available, with cost, turnaround time, sample quality and access identified as leading barriers.^7^ Methods that infer *EGFR* genotype from H&E WSIs offer a potential rapid path to mutation identification and initiation of treatment. Recently, 2 open-source artificial intelligence (AI)–powered models for *EGFR* prediction from H&E slides have been reported, each achieving high accuracy (AUCs near 0.85) in their external validations.^8,9^ In this study, we evaluated these 2 open-source models in 2 independent US and European cohorts and assess factors that may affect their performance.

## Methods

### Patients

The study included LUAD cohorts from Dana-Farber Cancer Institute (DFCI; June 2013 to November 2023), TNM-I (NCT03299478; August 2016 to February 2022), and TCGA LUAD included as technical validation dataset; all with paired next-generation sequencing data and H&E images. Mutation profiling used OncoPanel (DFCI) and TSO500 HT (TNM-I).^10,11^ Ancestry in DFCI was inferred using tumor-derived germline data.^12^ Image preprocessing and model inference followed the original EAGLE and DeepGEM reports for *EGFR* prediction.^8,9^ Both DFCI and TNM-I cohorts had ethics approval from their respective institutional review boards and written informed consent. The study followed the Transparent Reporting of a Multivariable Prediction Model for Individual Prognosis or Diagnosis (TRIPOD)–AI reporting guideline. *P* values <.05 were considered statistically significant. Methodological details are provided in the eMethods in Supplement 1. Data analysis took place from July 2025 to September 2025.

## Results

We first validated the reproducibility of the EAGLE and DeepGEM pipelines using the TCGA LUAD dataset (n = 463; *EGFR* mutation [12%]) with their original codebases and image-preprocessing workflows. The reproduced AUCs (0.89; 95% CI, 0.85-0.93 for EAGLE and 0.88; 95% CI, 0.82-0.93 for DeepGEM) matched the published results, ruling out concerns about technical misadaptation (eFigure 1 in Supplement 1).^8,9^ Overall, 2098 patients with LUAD were included (mean [SD] age, 66.6 [10.3] years; 1315 female individuals [63%] and 783 male individuals [37%]).

**Figure 1.**
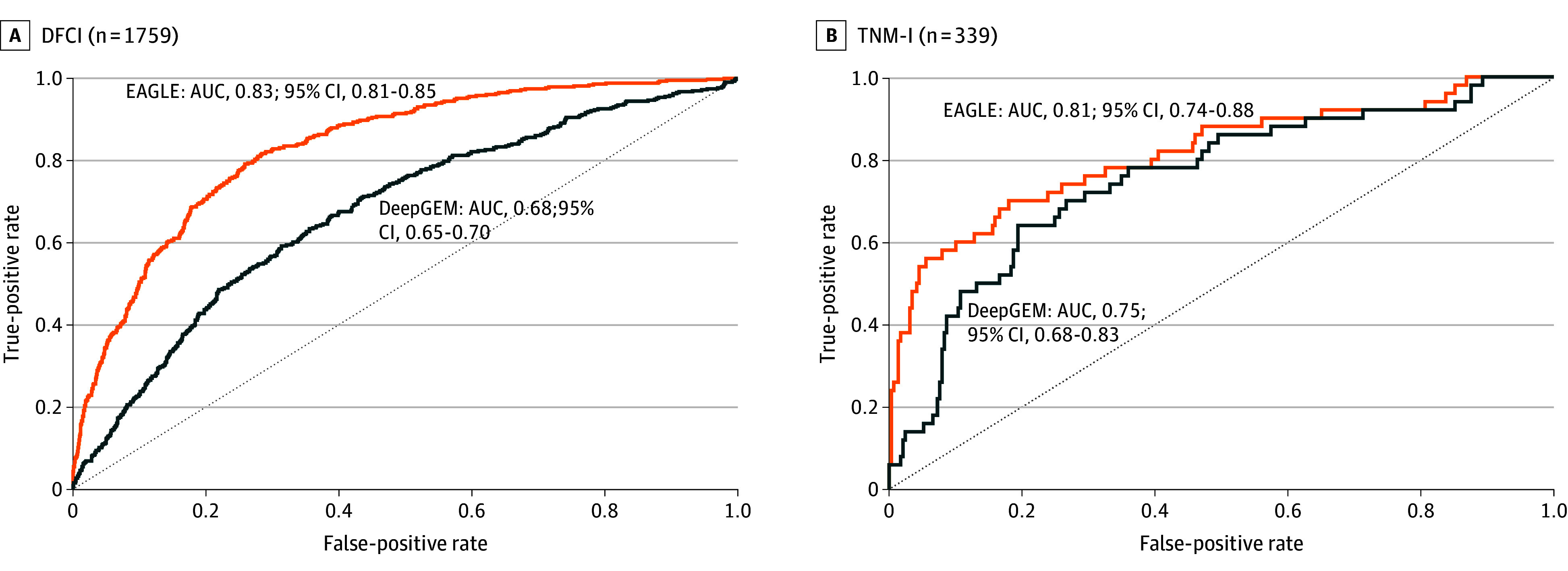
Receiver Operating Characteristic (ROC) Curves of Model Performance For *EGFR* Prediction ROC curves and AUCs for EAGLE vs DeepGEM, with 95% CIs calculated using 1000 bootstrap resamples. The dotted line indicates no discrimination (AUC = 0.5). AUC indicates area under the curve; DFCI, Dana-Farber Cancer Institute; TNM-I, the European-based trial.

Next, we deployed the models on the DFCI dataset (n = 1759). The cohort comprised 111 female individuals (63%), with median (range) age 67 (23-99) years, and with slides from a primary site in 1037 (60%) (eTable 1 in Supplement 1). *EGFR* mutations were present in 432 patients (25%), with exon 19 deletions most common (127 [29%]), followed by L858R substitutions (115 [27%]), with a wide variety of less common and double mutations (eTable 2 in Supplement 1). EAGLE showed an AUC of 0.83 (95% CI, 0.81-0.85) in this cohort vs 0.68 (95% CI, 0.65-0.70) for DeepGEM (Figure 1A). Notably, EAGLE probability scores did not differ significantly across *EGFR* mutation subtypes, supporting its robustness (*P* = .68; eMethods and eFigure 2 in Supplement 1).

We then evaluated both models in a secondary EU-based cohort, TNM-I (n = 339; eTable 1 in Supplement 1). *EGFR* mutations were detected in 50 patients (15%), most frequently exon 19 deletions (18 [36%]; eTable 3 in Supplement 1). In this cohort, EAGLE again outperformed DeepGEM with an AUC of 0.81 (95% CI, 0.74-0.88) vs 0.75 (95% CI, 0.68-0.83) (Figure 1B).

**Figure 2.**
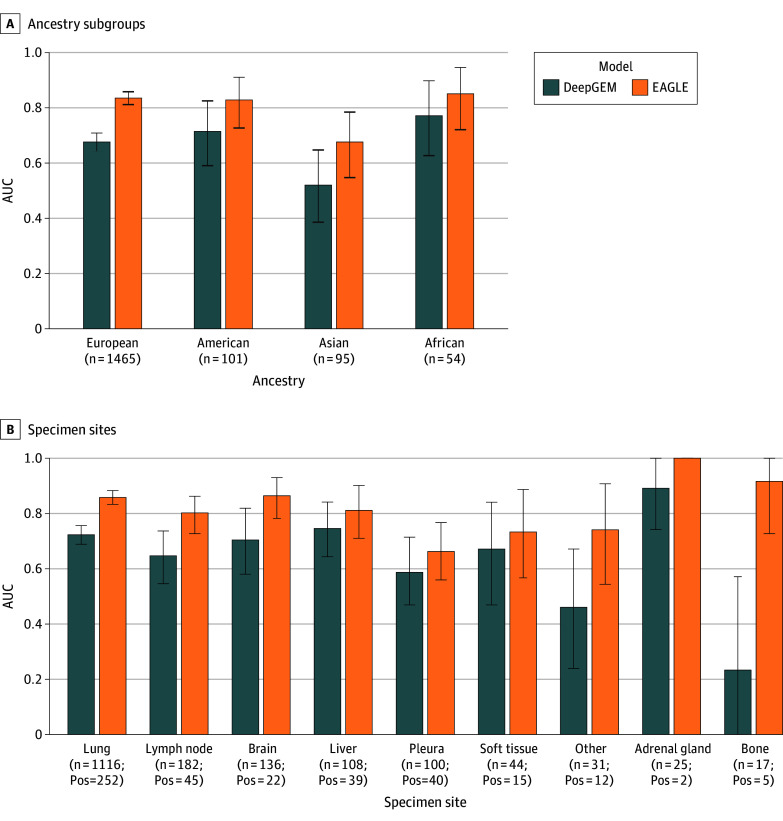
Bar Plots of Model Performance Across Ancestry and Specimen Sites Bar plots showing model performance across ancestry subgroups and specimen sites in the DFCI cohort. Error bars represent 95% CIs. Pos indicates the absolute count of cases of *EGFR* mutation in each group. AUC indicates area under the curve.

Previous studies have demonstrated the impact of demographics, where models trained predominantly on White patients may underperform in other populations.^13^ To explore this in our dataset, we examined the distribution of *EGFR* mutations across major genome-derived ancestral subgroups in the DFCI cohort. Cases of *EGFR* mutations were most frequent among Asian patients (60 [63%]), followed by African patients (17 [31%]) (eFigure 3 in Supplement 1). We evaluated both EAGLE and DeepGEM models across these ancestry subsets. EAGLE maintained robust performance (AUC, >0.80) across most groups, but its AUC dropped to 0.68 (95% CI, 0.55-0.78) in Asian patients (Figure 2A). This decline, despite the higher prevalence of *EGFR* mutations in this subgroup, may indicate sensitivity to morphological characteristics not fully captured during model training, or reflect underlying domain shifts in histopathologic presentation across populations. DeepGEM was inferior to EAGLE in all subgroups, including Asian patients (AUC, 0.52; 95% CI, 0.39-0.65 ), despite being originally trained on a Chinese population, highlighting the challenges of cross-cohort generalizability even in apparently similar ancestry groups (Figure 2A).

In addition, we assessed whether the high prevalence of *EGFR* mutations in the Asian subgroup could explain the observed decline in EAGLE performance. Under prevalence-matched subsampling, the Asian subgroup showed a mean AUC of 0.67 (95% CI, 0.53-0.82), compared with 0.83 (95% CI, 0.70-0.94) in the European subgroup (eFigure 4 in Supplement 1). This potentially suggests that the observed degradation in model performance in the Asian subgroup is not attributable to the higher prevalence of *EGFR* mutations.

Next, we evaluated model performance across primary vs metastatic tumor sample types in the DFCI cohort. Consistent with the original EAGLE report, performance decreased for metastatic samples compared with primary tumors (eFigure 5 in Supplement 1). We also assessed performance across the anatomical sites from which tumor samples were collected. EAGLE demonstrated strong and consistent performance across most specimen sites, except for pleural samples (n = 100), where the AUC dropped to 0.66 (Figure 2B). This decline suggests that pleural tissue may require particular attention during model development and validation.

Clinical application of such models relies on binarizing probability scores generated by the algorithms. Several thresholding approaches can be explored, with the most common being the Youden/optimal cutoff and the maximum F1 score.^14^ In our assessment for the DFCI cohort, the Youden index yielded a threshold of 0.05, while the F1-max method produced 0.12; accuracy was higher with the F1-based threshold (0.79) compared with the Youden threshold (0.75) (eFigure 6 in Supplement 1).

In line with the original EAGLE report,^8^ we adopted its dual-threshold cutoff system based on predicted probability scores to guide the decision of skipping rapid *EGFR* testing. If the score was below the negative predictive value (NPV) threshold, the case was classified as negative; if it was above the positive predictive value (PPV) threshold, the case was classified as positive. Cases with scores falling between these thresholds underwent *EGFR* (rapid) testing, enabling substantial test reduction while maintaining noninferior sensitivity and specificity compared with standard workflows. We applied this same cutoff strategy to our dataset (eFigure 7A in Supplement 1) with NPV threshold of 0.03 and PPV threshold of 0.95, achieving a maximum test reduction of 57% (eFigure 7B in Supplement 1) with sensitivity of 0.84, specificity of 0.99, PPV of 0.97, and NPV of 0.95 (eFigure 7C in Supplement 1).

## Discussion

This study provides one of the largest independent validations of open-source AI tools for *EGFR* mutation prediction in LUAD. These findings show that fine-tuned foundation models such as EAGLE maintained robust performance across US and European cohorts, whereas frozen feature-based approaches like DeepGEM degrade under domain shift. These findings underscore the importance of end-to-end model adaptation for clinical generalizability.^4,15^

A key observation was the variability in performance across patient subgroups. The accuracy of EAGLE was consistently high overall but declined in Asian patients and in pleural samples, despite higher *EGFR* mutation prevalence. This suggests that ancestry-related morphological variation and specimen context can influence algorithm behavior, highlighting the need for ancestry- and tissue-aware validation and calibration before deployment.

### Limitations

Limitations of this cohort study included restricting ancestry data into a single cohort, but the consistency of results across large US and European cohorts highlights the strong potential of AI-based pathologic analysis as a scalable complement to *EGFR* molecular testing.

## Conclusion

In this study, the dual-threshold strategy proposed for EAGLE translated well to both cohorts, enabling reduction of immediate molecular testing by up to 57% while maintaining high sensitivity and specificity, with the option to select higher-sensitivity thresholds at the cost of minimal test reduction. Such triage could accelerate treatment initiation and reduce molecular assay burden, particularly in resource-limited settings.

## Notes

Supplement 1. eMethods eFigure 1. Model performance in TCGA lung adenocarcinoma (LUAD) eFigure 2. Distribution of EAGLE probability scores (PS) across different EGFR variant subtypes in DFCI cohort eFigure 3. EGFR mutation frequency eFigure 4. EGFR prevalence-matched subsampling analysis across ancestry subgroups eFigure 5. Model performance across tumor types eFigure 6. Threshold optimization and performance evaluation of EAGLE for EGFR mutation binary prediction in the DFCI cohort eFigure 7. EAGLE triage thresholds on the DFCI cohort (n=1759) eTable 1. Clinical characteristics of the lung adenocarcinoma cohorts used to validate the models eTable 2. Distribution of EGFR mutated subtypes in the DFCI (n=1759) cohort eTable 3. Distribution of EGFR mutated subtypes in the TNM-I (n=339) cohort

Supplement 2. Data Sharing Statement
