# Supplementary material for: Ancestry-Associated Performance Variability of Open-Source AI Models for EGFR Prediction in Lung Cancer
Source: JAMA Oncol. 2026 Feb 12;12(4):402–6. doi: 10.1001/jamaoncol.2025.6430 (PMC12902924; doi:10.1001/jamaoncol.2025.6430)
Supplement: Supplement 1. — eMethods eFigure 1. Model performance in TCGA lung adenocarcinoma (LUAD) eFigure 2. Distribution of EAGLE probability scores (PS) across different EGFR variant subtypes in DFCI cohort eFigure 3. EGFR mutation frequency eFigure 4. EGFR prevalence-matched subsampling analysis across ancestry subgroups eFigure 5. Model performance across tumor types eFigure 6. Threshold optimization and performance evaluation of EAGLE for EGFR mutation binary prediction in the DFCI cohort eFigure 7. EAGLE triage thresholds on the DFCI cohort (n=1759) eTable 1. Clinical characteristics of the lung adenocarcinoma cohorts used to validate the models eTable 2. Distribution of EGFR mutated subtypes in the DFCI (n=1759) cohort eTable 3. Distribution of EGFR mutated subtypes in the TNM-I (n=339) cohort [file jamaoncol-e256430-s001.pdf]

# Supplemental Online Content

Rakaee M, Nassar AH, Tafavvoghi M, et al. Ancestry-associated performance variability of open-source AI models for *EGFR* prediction in lung cancer. *JAMA Oncol*. Published online February 12, 2026. doi:10.1001/jamaoncol.2025.6430

**eMethods**
**eFigure 1.** Model performance in TCGA lung adenocarcinoma (LUAD)
**eFigure 2.** Distribution of EAGLE probability scores (PS) across different EGFR variant subtypes in DFCI cohort
**eFigure 3.** EGFR mutation frequency
**eFigure 4.** EGFR prevalence-matched subsampling analysis across ancestry subgroups
**eFigure 5.** Model performance across tumor types
**eFigure 6.** Threshold optimization and performance evaluation of EAGLE for EGFR mutation binary prediction in the DFCI cohort
**eFigure 7.** EAGLE triage thresholds on the DFCI cohort (n=1759)
**eTable 1.** Clinical characteristics of the lung adenocarcinoma cohorts used to validate the models
**eTable 2.** Distribution of EGFR mutated subtypes in the DFCI (n=1759) cohort
**eTable 3.** Distribution of EGFR mutated subtypes in the TNM-I (n=339) cohort

This supplemental material has been provided by the authors to give readers additional information about their work.

© 2026 Rakaee M et al. *JAMA Oncol*.

# eMethods

## *Patients and ethics*

*DFCI cohort*: Between June 2013 and November 2023, 4887 patients with LUAD at Dana-Farber Cancer Institute (DFCI) underwent OncoPanel next-generation sequencing (NGS). Of these, 1761 had paired NGS data and H&E-stained WSIs available. Two cases were excluded due to poor image quality, leaving 1759 patients for analysis.

*TNM-I cohort*: 851 patients with early-stage, resectable NSCLC were prospectively enrolled from five centers in Norway and Denmark between August 2016 and February 2022 (NCT03299478). Of these, 502 underwent NGS analysis, and 339 were histologically confirmed as LUAD. Both cohorts were approved by local ethics committees (DFCI: #25-631 & #2021P000557; TNM-I: REK2016/2054) and all patients provided informed consent.

For technical reproducibility, TCGA LUAD data were obtained from the publicly accessible NCI-GDC repository. In total, 463 unique patients had paired genomic profiling data and corresponding H&E images.

## *EGFR* mutation calling

In the DFCI cohort, genetic analysis was routinely performed using the OncoPanel NGS assay, implemented in three successive versions targeting 275, 300, and 447 cancer-associated genes. This is a CLIA-certified platform, with internally developed bioinformatics pipeline [1,2]. *EGFR* variants were re-annotated using the OncoKB Annotator API (v3.4) [3]. In the TNM-I cohort, genetic analysis was performed with the TruSight Oncology 500 HT NGS assay (TSO500; 523 genes; tumor-only)[4]. Alignment and variant calling were conducted with Mutect2-GATK (v2.2)[5], followed by annotation and classification using PCGR (v2.1.2)[6]. In both cohorts, variants classified as oncogenic or likely oncogenic within the kinase domain (exons 18-24) were considered *EGFR*-mutated (positive)[7].

## Ancestry calling

The method has been described in detail previously [8]. Briefly, for the DFCI cohort, we constructed an ancestry reference panel by merging germline genotype data from the 1000 Genomes Project, TCGA normal samples, and the Partners Biobank, restricting the dataset to TCGA-typed variants and applying LD pruning. Ancestry inference was performed using

PLINK fastPCA. The principal components captured continental population structure (European, African, East Asian, South Asian and American), and the inclusion of Partners Biobank samples improved representation of admixed individuals [8,9]. South Asian (n=19) and East Asian (n=76) are combined for subgroup anlaysis.

**Slide digitalization**

In the DFCI cohort, the same hematoxylin and eosin (H&E) slides used for NGS were digitized at a resolution of 0.49 µm/pixel using an Aperio ScanScope AT system (Leica Biosystems; svs format). In the TNM-I cohort, paired H&E slides corresponding to lung adenocarcinoma cases analyzed by NGS were scanned at a resolution of 0.25 µm/pixel using a Pannoramic 250 Flash III scanner (3DHISTECH; mrxs format).

**Model inference**

For EAGLE, whole slide images were tiled into 224 x 224 px tiles at 20x (0.5 µm/px) for the tile encoder. The encoder was a fine-tuned version of the Prov-GigaPath foundation model (ViT-g) and produced 1536-dimensional patch features[10]. A gated multiple instance learning (MIL) attention mechanism aggregated all patch embeddings into a slide-level representation for *EGFR* classification. For DeepGEM, whole slide images were tiled into 1120 x 1120 px tiles at 20x. Patch features (768-D) were extracted using a self-supervised CTransPath encoder[11]. A custom transformer-based MIL aggregator then generated the final whole slide-level predictions for *EGFR* status. Both models were evaluated on the TCGA, DFCI and TNM-I cohorts using Ubuntu Linux (v.20) with NVIDIA A100 GPU. Full methodological details for both pipelines are described in their original publications[7,12].

**Statistical analysis**

Statistical analyses were performed in Python using relevant libraries. Receiver operating characteristic (ROC) analysis was used to calculate area under the curve (AUC), sensitivity (recall), specificity, F1 score, accuracy, precision (positive predictive value; PPV), and negative predictive value (NPV) for *EGFR* probability scores. Confidence intervals were estimated using 1000 bootstrap resamples. *EGFR* probability scores were categorized using the Youden index and F1-max. Group differences in EAGLE probability scores across *EGFR* variant subtypes were assessed with the Kruskal–Wallis test followed by Dunn's post-hoc comparisons. For prevalence-matched subsampling, we balanced *EGFR* mutation prevalence

across subgroups to the overall DFCI cohort prevalence (25%) using Monte Carlo sampling procedure. In each iteration, 40 cases (limited by smaller negative class in the Asian subgroup) were drawn per subgroup. This process was repeated 250 times, AUC was calculated for each iteration, and mean AUC with 95% confidence intervals was reported, comparing the Asian vs. European subgroup. Dual-threshold triage was implemented as described in the original EAGLE report, comparing NPV/PPV to the rapid assay used in that study (Idylla) [7]. Briefly, to determine the NPV and PPV thresholds a range of candidate threshold pairs was evaluated, and for each pair we assessed PPV, NPV and the proportion of rapid tests that would be avoided. Thresholds that maintained PPV and NPV at least as high as the reference rapid assay (Idylla: NPV=0.954, PPV=0.988) were considered noninferior. Among these, the thresholds that maximized rapid-test reduction were selected.

**Additional information**

The EAGLE and DeepGEM codes are publicly available from their original publications:

EAGLE: https://github.com/chadvanderbilt/EAGLE

DeepGEM: https://github.com/TencentAILabHealthcare/DeepGEM

Model weights

EAGLE: https://huggingface.co/MCCPBR/EAGLE/tree/main

DeepGEM:
https://github.com/TencentAILabHealthcare/DeepGEM/tree/main/checkpoints/DeepGEM

**Extended References:**


1. Sholl LM, Do K, Shivdasani P, et al. Institutional implementation of clinical tumor profiling on an unselected cancer population. *JCI Insight*. 2016;1(19):e87062. doi:10.1172/jci.insight.87062
2. Ricciuti B, Wang X, Alessi J V., et al. Association of High Tumor Mutation Burden in Non-Small Cell Lung Cancers With Increased Immune Infiltration and Improved Clinical Outcomes of PD-L1 Blockade Across PD-L1 Expression Levels. *JAMA Oncol*. 2022;8(8):1160-1168. doi:10.1001/JAMAONCOL.2022.1981
3. Suehnholz SP, Nissan MH, Zhang H, et al. Quantifying the Expanding Landscape of Clinical Actionability for Patients with Cancer. *Cancer Discov*. 2024;14(1):49-65. doi:10.1158/2159-8290.CD-23-0467
4. Rakaee M, Andersen S, Giannikou K, et al. Machine learning-based immune phenotypes correlate with STK11/KEAP1 co-mutations and prognosis in resectable

NSCLC: a sub-study of the TNM-I trial. *Ann Oncol*. 2023;34(7):578-588. doi:10.1016/j.annonc.2023.04.005
5. McKenna A, Hanna M, Banks E, et al. The genome analysis toolkit: A MapReduce framework for analyzing next-generation DNA sequencing data. *Genome Res*. 2010;20(9):1297-1303. doi:10.1101/gr.107524.110
6. Nakken S, Fournous G, Vodák D, Aasheim LB, Myklebost O, Hovig E. Personal Cancer Genome Reporter: Variant interpretation report for precision oncology. *Bioinformatics*. 2018;34(10):1778-1780. doi:10.1093/bioinformatics/btx817
7. Campanella G, Kumar N, Nanda S, et al. Real-world deployment of a fine-tuned pathology foundation model for lung cancer biomarker detection. *Nat Med*. Published online 2025. doi:10.1038/s41591-025-03780-x
8. Nassar AH, Adib E, Abou Alaiwi S, et al. Ancestry-driven recalibration of tumor mutational burden and disparate clinical outcomes in response to immune checkpoint inhibitors. *Cancer Cell*. 2022;40(10):1161-1172.e5. doi:10.1016/j.ccell.2022.08.022
9. Galinsky KJ, Bhatia G, Loh PR, et al. Fast Principal-Component Analysis Reveals Convergent Evolution of ADH1B in Europe and East Asia. *Am J Hum Genet*. 2016;98(3):456-472. doi:10.1016/j.ajhg.2015.12.022
10. Xu H, Usuyama N, Bagga J, et al. A whole-slide foundation model for digital pathology from real-world data. *Nature*. 2024;630(8015):181-188. doi:10.1038/S41586-024-07441
11. Wang X, Yang S, Zhang J, et al. Transformer-based unsupervised contrastive learning for histopathological image classification. *Med Image Anal*. 2022;81:102559. doi:10.1016/J.MEDIA.2022.102559
12. Zhao Y, Xiong S, Ren Q, et al. Deep learning using histological images for gene mutation prediction in lung cancer: a multicentre retrospective study. *Lancet Oncol*. 2025;26(1):136-146. doi:10.1016/S1470-2045(24)00599-0

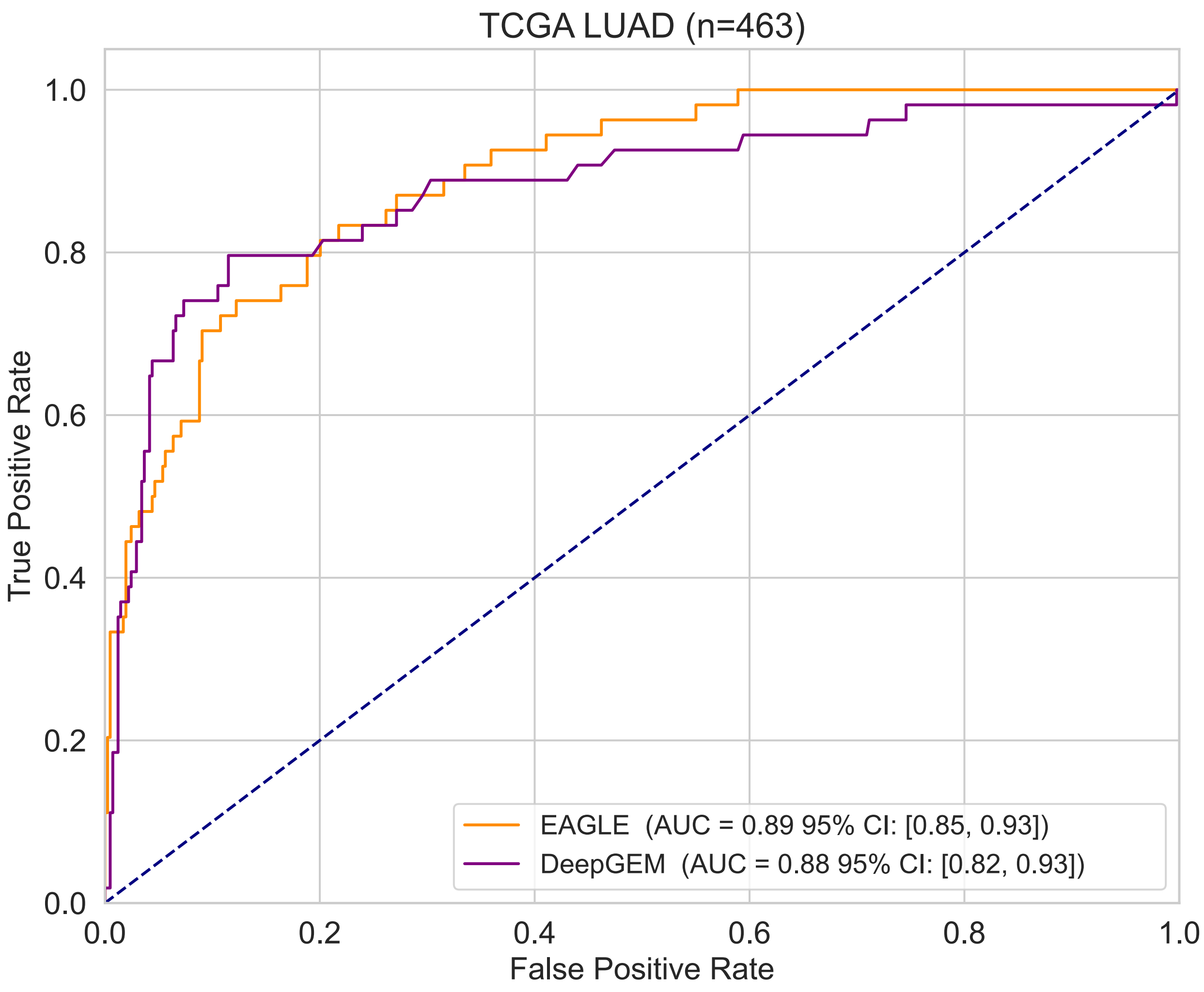


**eFigure 1: Model performance in TCGA lung adenocarcinoma (LUAD).** ROC curves and AUCs for EAGLE vs. DeepGEM, with 95% confidence intervals calculated using 1000 bootstrap resamples.

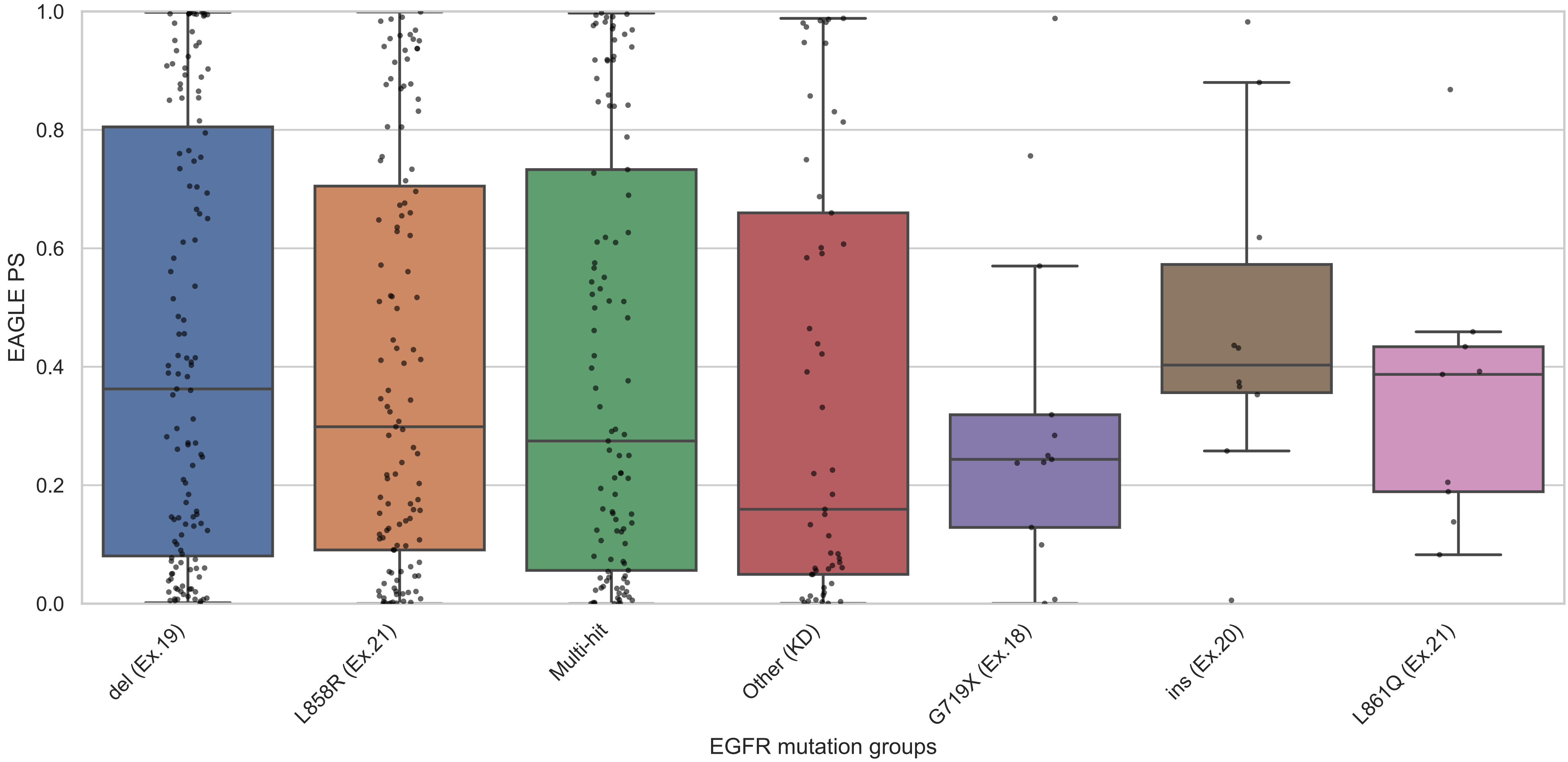


**eFigure 2: Distribution of EAGLE probability scores (PS) across different EGFR variant subtypes in DFCI cohort**. *Multi-hit* refers to any case with more than one EGFR-altered variant, as listed in **eTable 2**. KD, kinase domain; Ex, exon; PS, probability score.

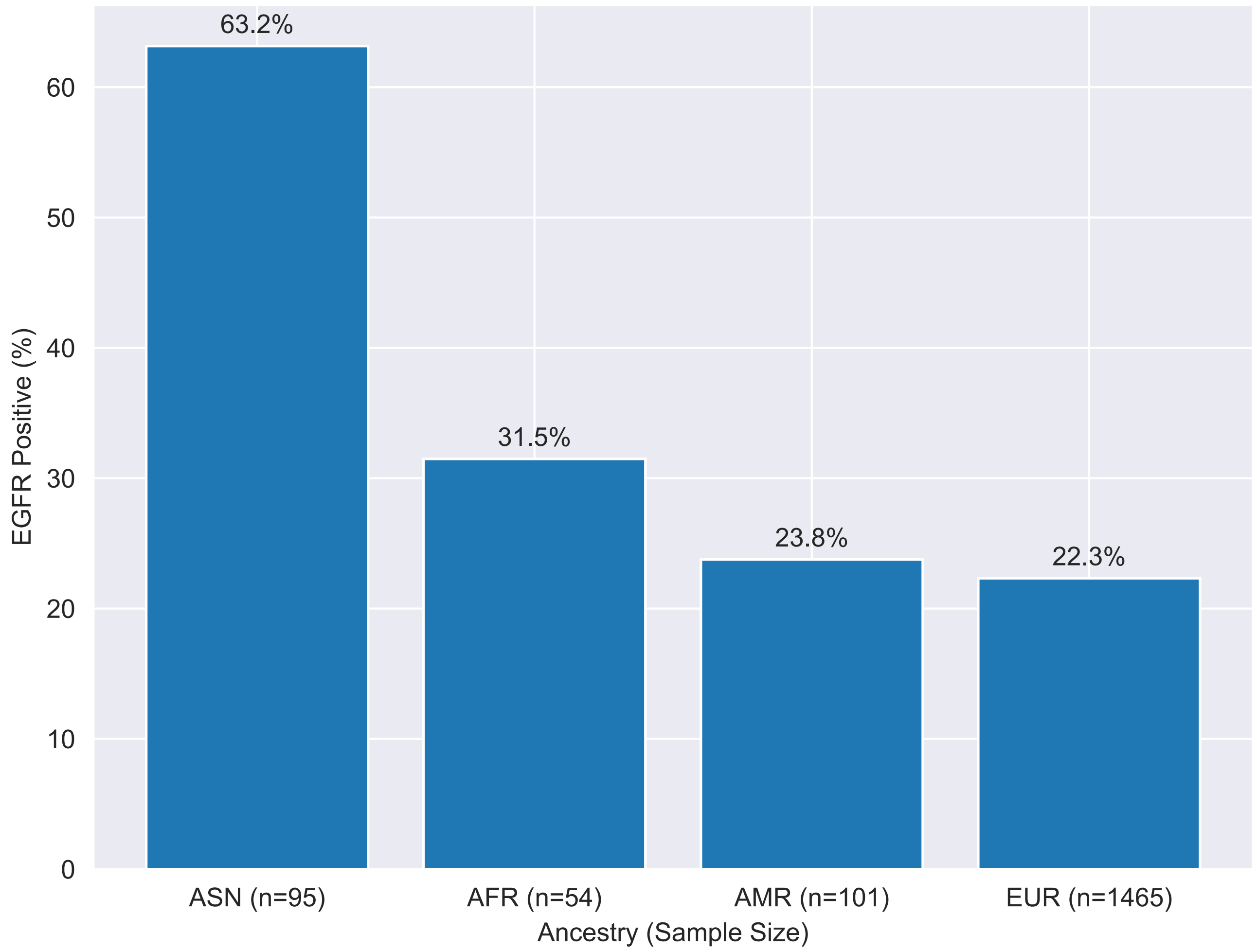


**eFigure 3: EGFR mutation frequency**. Bar plots showing the frequency of EGFR mutations across ancestry subgroups in the DFCI cohort (n=1715). Cases with unknown ancestry (n=44) are excluded.

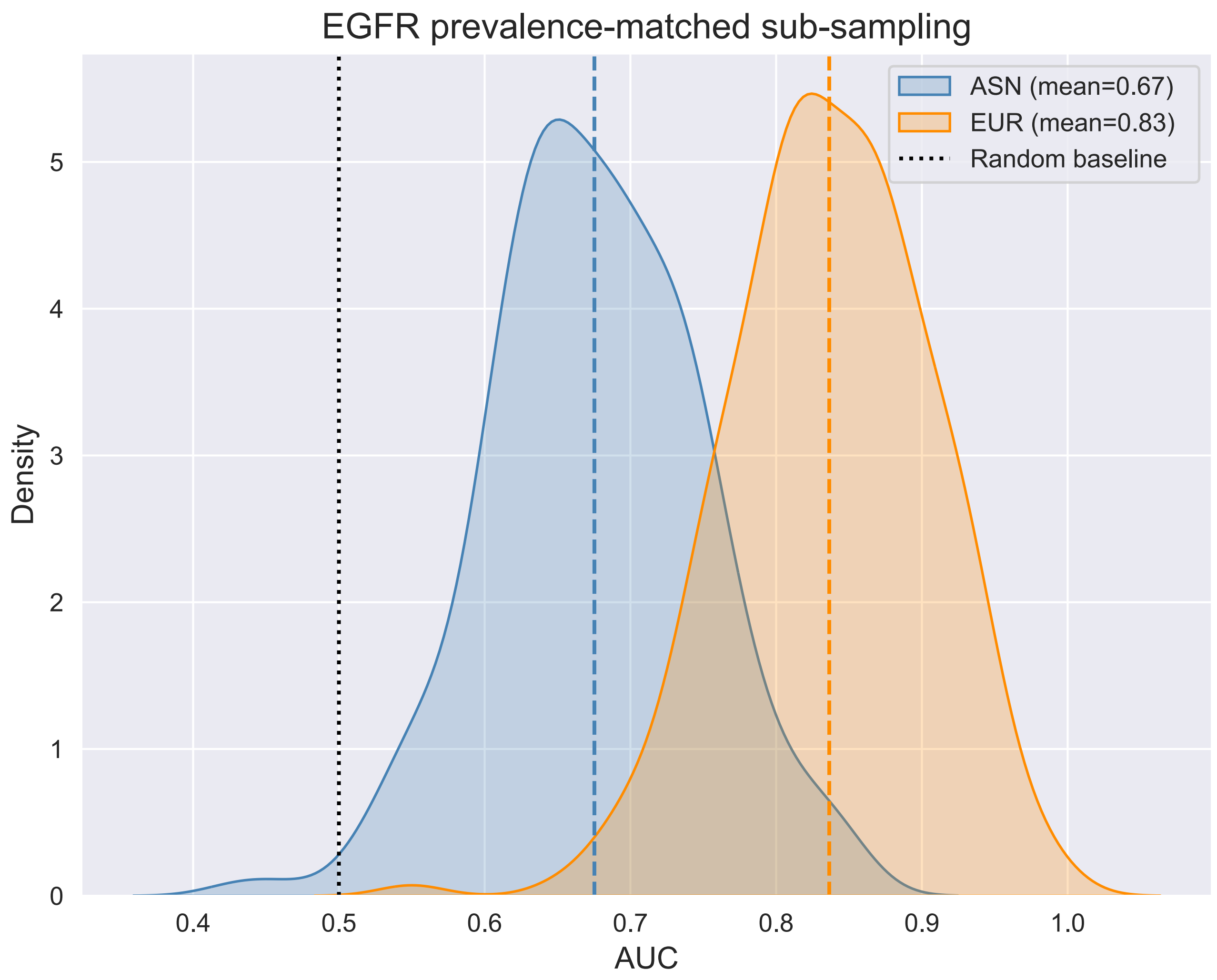


**eFigure 4: EGFR prevalence-matched subsampling analysis across ancestry subgroups**: Density distributions of AUC values from 250 subsampling iterations of the EAGLE model in Asian (ASN) and European (EUR) subgroups of the DFCI cohort. Dash lines indicate mean and baseline.

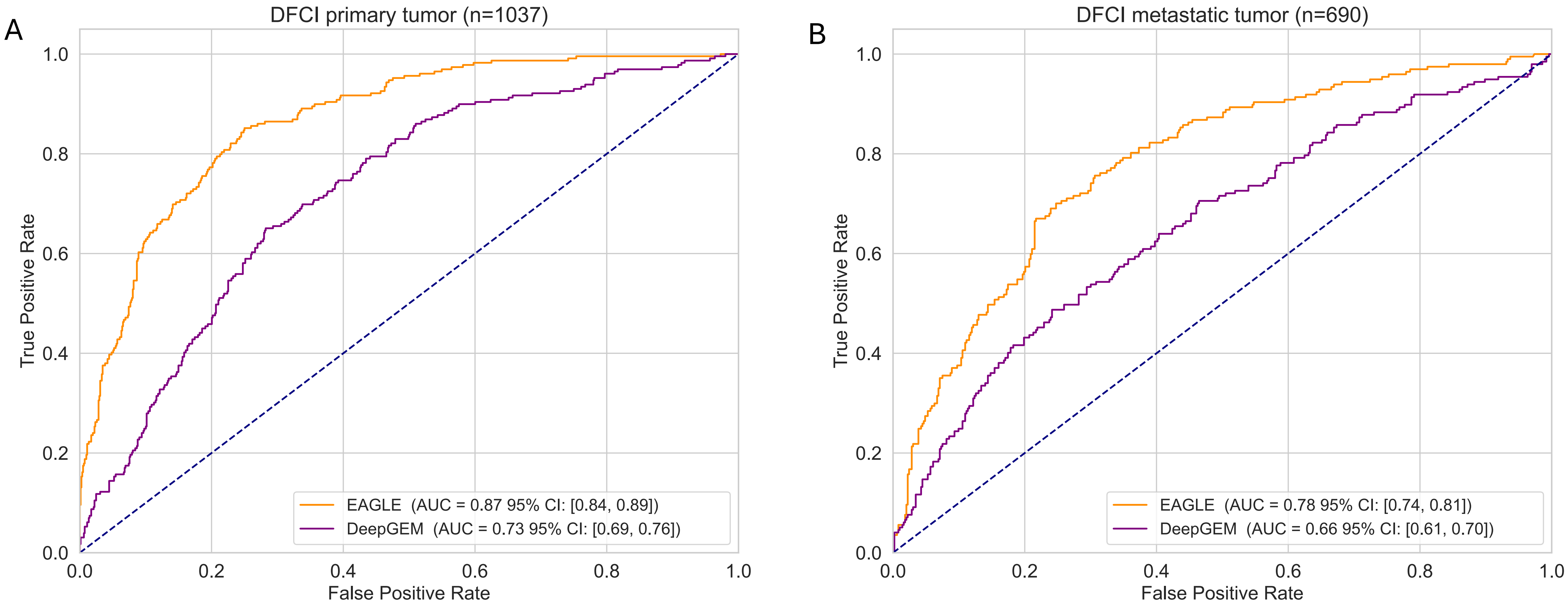


**eFigure 5: Model performance across tumor types**. A) primary tumors. B) Metastatic tumors.

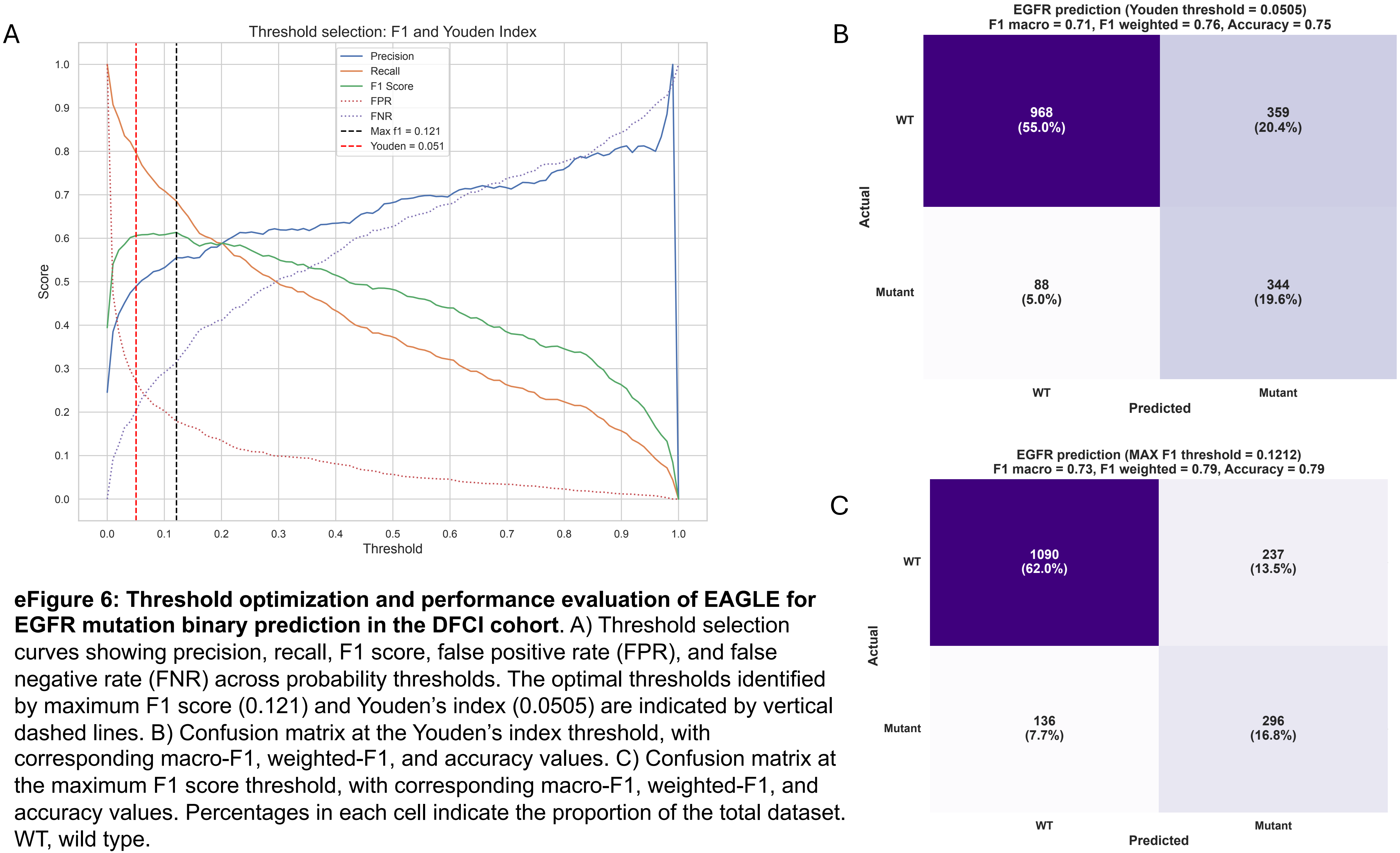


**eFigure 6: Threshold optimization and performance evaluation of EAGLE for EGFR mutation binary prediction in the DFCI cohort**. A) Threshold selection curves showing precision, recall, F1 score, false positive rate (FPR), and false negative rate (FNR) across probability thresholds. The optimal thresholds identified by maximum F1 score (0.121) and Youden's index (0.0505) are indicated by vertical dashed lines. B) Confusion matrix at the Youden's index threshold, with corresponding macro-F1, weighted-F1, and accuracy values. C) Confusion matrix at the maximum F1 score threshold, with corresponding macro-F1, weighted-F1, and accuracy values. Percentages in each cell indicate the proportion of the total dataset. WT, wild type.

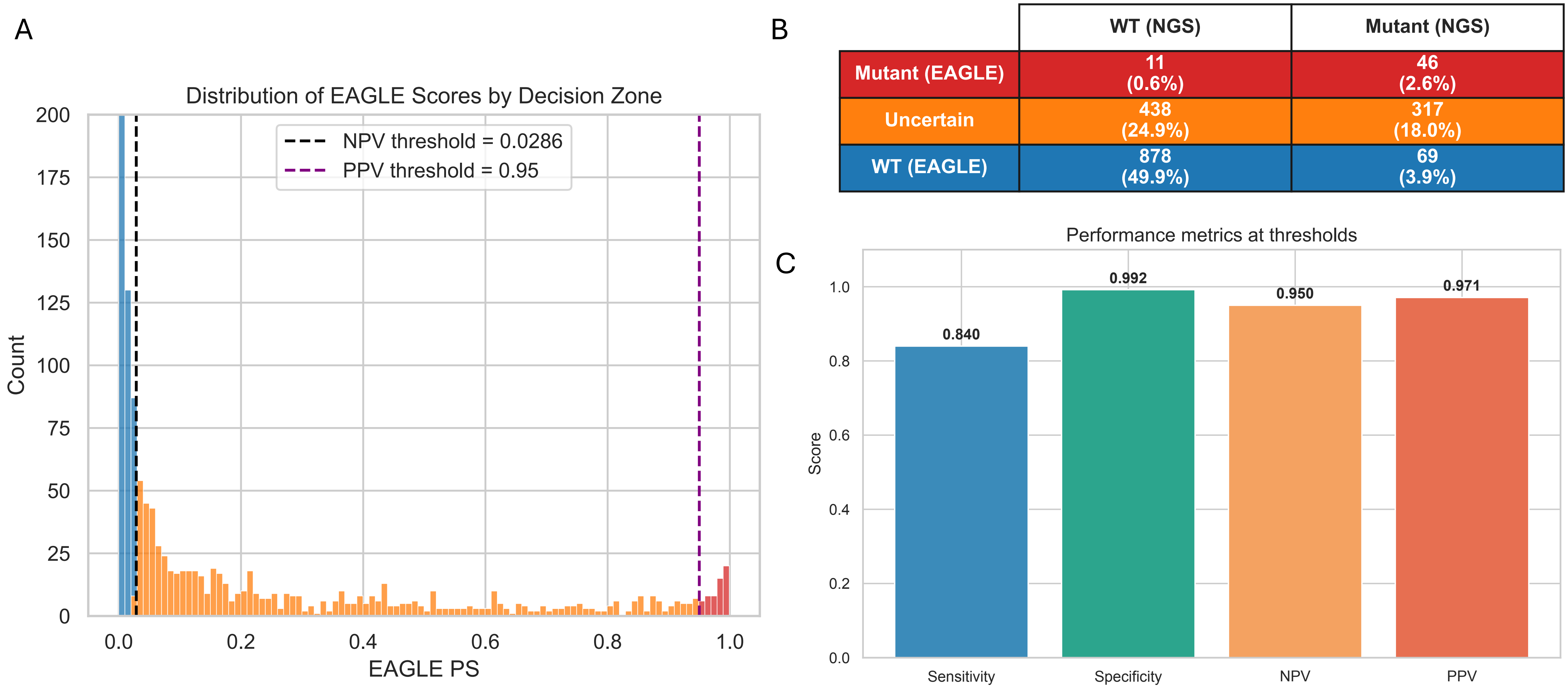


| | WT (NGS) | Mutant (NGS) |
|---|---|---|
| Mutant (EAGLE) | 11 (0.6%) | 46 (2.6%) |
| Uncertain | 438 (24.9%) | 317 (18.0%) |
| WT (EAGLE) | 878 (49.9%) | 69 (3.9%) |

**eFigure 7 : EAGLE triage thresholds on the DFCI cohort (n=1759). A)** Histogram of EAGLE per-slide probabilities (PS) with the negative-predictive-value (NPV) cut-off (dashed black) and positive-predictive-value (PPV) cut-off (dashed purple). # of bin=100. **B)** Color-coded triage table (rows = EAGLE PS groups; columns = NGS ground truth). Cells report absolute case counts with overall cohort percentages in parentheses. **C)** Bar plot of performance at these thresholds (n=1004).

**eTable 1:** Clinical characteristics of the lung adenocarcinoma cohorts used to validate the models.

| | **DFCI cohort N = 1759 (%)** [a] | **TNM-I cohort N = 339 (%)** |
|---|---|---|
| **Age** | | |
| **median (range)** | 67 (23-99) | 69 (44-84) |
| | | |
| **Gender** | | |
| **female** | 1111 (63) | 204 (60) |
| **male** | 648 (37) | 135 (40) |
| **Smoking** | | |
| **Former** | 820 (60) | 205 (61) |
| **current** | 186 (14) | 96 (28) |
| **never** | 349 (26) | 38 (11) |
| **unknown** | 404 | |
| **Specimen site** | | |
| **lung** | 1116 (63) | 339 (100) |
| **lymph node** | 182 (10) | |
| **brain** | 136 (8) | |
| **liver** | 108 (6) | |
| **pleura** | 100 (6) | |
| **soft tissue** | 44 (3) | |
| **other** [b] | 31 (2) | |
| **adrenal gland** | 25 (1) | |
| **bone** | 17 (1) | |
| **Tumor type** | | |
| **primary** | 1037 (60) | 339 (100) |
| **metastatic** | 690 (40) | |
| **unknown** | 32 | |

[a], Percentages exclude unknown/missing values.
[b], other includes peritoneum, kidney, skin, head and neck, breast, stomach and small intestine.

**eTable 2**: Distribution of EGFR mutated subtypes in the DFCI (n=1759) cohort

| EGFR subtypes | Count | % |
|---|---|---|
| del (Ex.19) | 127 | 29.40 |
| L858R (Ex.21) | 115 | 26.62 |
| Other (KD) | 53 | 12.27 |
| G719X (Ex.18); Other (KD) | 22 | 5.09 |
| L858R (Ex.21); Other (KD) | 19 | 4.40 |
| T790M (Ex.20); del (Ex.19) | 19 | 4.40 |
| L858R (Ex.21); T790M (Ex.20) | 15 | 3.47 |
| G719X (Ex.18) | 13 | 3.01 |
| ins (Ex.20) | 10 | 2.31 |
| L861Q (Ex.21) | 9 | 2.08 |
| Other (KD); del (Ex.19) | 8 | 1.85 |
| L858R (Ex.21); Other (KD); T790M (Ex.20) | 8 | 1.85 |
| Other (KD); T790M (Ex.20); del (Ex.19) | 5 | 1.16 |
| L861Q (Ex.21); Other (KD) | 4 | 0.93 |
| L861Q (Ex.21); T790M (Ex.20) | 2 | 0.46 |
| G719X (Ex.18); L861Q (Ex.21) | 1 | 0.23 |
| Other (KD); T790M (Ex.20) | 1 | 0.23 |
| Other (KD); ins (Ex.20) | 1 | 0.23 |
| Total | 432 | 100 |

Abbrevations: Ex, exon; del, deletion; ins, insertion; KD, kinase domain
Other includes p.C797S, p.D761Y, p.D770_N771insG, p.D770_N771insN, p.D770_N771insNH, p.D770_N771insNPG, p.D770_N771insNPH, p.E709A, p.E709K, p.E709V, p.E746_P753delinsVS, p.E746_R748del, p.E804K, p.F747_S753delinsL, p.G917R, p.H773L, p.I740_K745dup, p.I744M, p.I744_A750delinsVK, p.K754E, p.K757M, p.L718Q, p.L747A, p.L747P, p.L747_A755delinsAT, p.L747_A755delins, p.L747_R750delinsA, p.L747_S752del, p.L792H,p.L833F, p.L833V,p.L838V, p.L858M,p.L861R, p.N771_P772insH, p.N771_P772ins, p.N771_P772insPH, p.N771delinsTH, p.N771delinsVH, p.P772_H773insH, p.P772_H773insHA, p.P772_H773insHT, p.R776H, p.S752_I759del, p.S768I, p.S768_D770dup, p.T751_E758del, p.T790_C797delins, p.V769L, p.V774M, p.V834L.

**eTable 3**: Distribution of EGFR mutated subtypes in the TNM-I (n=339) cohort

| EGFR subtypes | Count | % |
|---|---|---|
| del (Ex.19) | 18 | 36 |
| L858R (Ex.21) | 10 | 20 |
| Other (KD) | 7 | 14 |
| ins (Ex.20) | 7 | 14 |
| del (Ex.19); Other (KD) | 2 | 4 |
| G719X (Ex.18) | 1 | 2 |
| L858R (Ex.21); Other (KD) | 1 | 2 |
| L861Q (Ex.21) | 1 | 2 |
| L861Q (Ex.21); Other (KD) | 1 | 2 |
| Other (KD); ins (Ex.20) | 1 | 2 |
| T790M (Ex.20); Other (KD) | 1 | 2 |
| Total | 50 | 100 |

Abbrevations: Ex, exon; del, deletion; ins, insertion; KD, kinase domain
Other includes p.Ala743Thr, p.Ala750_Thr751insP, p.Gln812Pro, p.Glu746_Ala750del, p.Glu922Ter, p.Gly724=, p.Gly779Cys, p.Ile744Phe, p.Leu747Ter, p.Leu760ProfsTer3, p.Leu814_Leu815insProTer, p.Leu858Arg, p.Leu861Gln, p.Phe795=, p.Ser768=, p.Ser768Ile, p.Thr751AspfsTer23, p.Thr790Met, p.Tyr727CysfsTer22, p.Val769Leu.
