# Supplementary material for: Ancestry-Associated Performance Variability of Open-Source AI Models for EGFR Prediction in Lung Cancer
Source: JAMA Oncol. 2026 Feb 12;12(4):402–6. doi: 10.1001/jamaoncol.2025.6430 (PMC12902924; doi:10.1001/jamaoncol.2025.6430)
Supplement: Supplement 2. — Data Sharing Statement [file jamaoncol-e256430-s002.pdf]

Data Sharing Statement

Rakaee. Ancestry-Associated Performance Variability of Open-Source AI Models for EGFR Prediction in Lung Cancer. *JAMA Oncol*. Published February 12, 2026.
doi:10.1001/jamaoncol.2025.6430

Data
Data available: No

Additional Information
Explanation for why data not available: Requests for sharing the clinical data should be directed to the corresponding author by non-commercial entities and must be reasonable. Due to the multi-centre nature of the study, data sharing will require approval from the principal investigator at each contributing center.
